# Long noncoding RNA SNHG20 promotes gastric cancer progression by inhibiting p21 expression and regulating the GSK-3β/ β-catenin signaling pathway

**DOI:** 10.18632/oncotarget.20959

**Published:** 2017-09-16

**Authors:** Jie Liu, Lanyu Liu, Jin-Xiang Wan, Ying Song

**Affiliations:** ^1^ Department of Intensive Care Unit, Renmin Hospital, Hubei University of Medicine, Shiyan, Hubei, China; ^2^ Department of Gynecology and Obstetrics, Weifang Hospital of Maternal and Child Health, Weifang, Shandong Province, China; ^3^ Department of Medical Ultrasonics, Huai'an Second People's Hospital, The Affiliated Huai'an Hospital of Xuzhou Medical University, Huai'an, China; ^4^ Department of Outpatient, People's Hospital of Zoucheng, Zoucheng, China

**Keywords:** gastric cancer, small nucleolar RNA host gene 20, EZH2, p21

## Abstract

Recent studies have indicated that long non-coding RNAs (lncRNAs) play important regulatory roles in tumor development and progression. However, the contribution of small nucleolar RNA host gene 20 (SNHG20) to gastric cancer development remains largely unknown. The aim of the study is to investigate the functional significance of SNHG20 involved in gastric cancer (GC) progression. In the study, our results demonstrated that the expression levels of SNHG20 were remarkably up-regulated in GC cells. Functionally, SNHG20 promoted the GC MKN45 and BGC-823 cells proliferation and invasion. Furthermore, knockdown of SNHG20 significantly inhibited the epithelial-mesenchymal transition (EMT) in MKN45 and BGC-823 cells, whereas, the overexpression of SNHG20 had the promoting effects. Moreover, we found that overexpression of SNHG20 in MKN45 and BGC-823 cells significantly inhibited the expression of E-cadherin and p21 via binding to EZH2 and regulated the GSK-3β/β-catenin signaling pathway. Thus, the results showed that SNHG20 acted as an oncogene in GC and targeting SNHG20 may serve as a therapeutic target for GC.

## INTRODUCTION

Gastric cancer (GC) is one of the most common digestive malignant tumors and ranks as the second cause of death by malignancy worldwide [1, 2]. About 35% GC patients appear synchronous distant metastases and the vast majority of patients present with hepatic metastatic disease, sometimes accompanied by synchronous peritoneal and lung dissemination [3]. In the recent years, survival of GC patients has shown only minor improvement, although significant advances including diagnostic techniques, surgical and chemotherapeutic approaches, the development of novel therapeutic agents [4]. Therefore, an improved understanding of molecular biology mechanisms involved in gastric cancer progression will facilitate the development of effective targeted therapeutic strategies for patients.

Long non-coding RNAs (LncRNAs) have attracted much attention in cancer research field and act as key regulators of cellular development, proliferation, differentiation and apoptosis. The dysregulation of LncRNAs has been reported in tumor initiation, progression, invasion and metastasis of gastric cancer [5, 6]. For example, the overexpression of HOTAIR predicted a poor prognosis in GC patients, and HOTAIR could epigenetically silence miR-34a by binding to PRC2 to promote the epithelial-to-mesenchymal transition (EMT) [7]. Kong *et al*. found long non-coding RNA PVT1 indicated a poor prognosis in gastric cancer patients and promoted GC cell proliferation through epigenetically regulating p15 and p16 expression [8]. Zhao *et al*. reported that long non-coding RNA Linc00152 was involved in cell cycle arrest, apoptosis, epithelial to mesenchymal transition, cell migration and invasion in gastric cancer [9]. Aberrant expression of MALAT1 was confirmed to bind EZH2, suppressed the tumor suppressor PCDH10, and promoted gastric cancer cell migration and invasion [10].

Small nucleolar RNA host gene 20 located at 17q25.2 was originally reported in hepatocellular carcinoma (HCC) and promoted the cell proliferation [11]. Another study confirmed that SNHG20 expression was significantly up-regulated in colorectal cancer (CRC) and knockdown of SNHG20 suppressed CRC cell proliferation, invasion and migration, and cell cycle progression [12]. To date, nothing has been known how SNHG20 are involved in GC progression. Therefore, we conducted this study to investigate the functional significance of SNHG20 in gastric cancer (GC) progression and to explore possible mechanisms underlying its functionality.

## MATERIALS AND METHODS

### Cell culture

Three human GC cell lines (BGC823, SGC-7901 and MKN45) and an immortalized normal gastric epithelial cell line GES-1 were purchased from the Institute of Biochemistry and Cell Biology of the Chinese Academy of Sciences (Shanghai, China). All cells were cultured in RPMI 1640 medium (GIBCO, Carlsbad, CA) supplemented with 10% fetal bovine serum (FBS), 100 U/ml penicillin and 100 mg/ml streptomycin (Invitrogen, Carlsbad, CA, USA) in humidified air at 37°C with 5% CO2.

### RNA isolation and qRT-PCR

Total RNA was extracted from the cells by using Trizol reagent (TaKaLa, Dalian, China), according to the manufacturer's instructions. RNA was reverse transcribed to cDNA by using a Reverse Transcription Kit (Takara, Dalian, China). Real-time PCR analysis was carried out with SYBR Premix Ex Taq (Takara, Dalian China). Results were normalized to the expression of GAPDH. The cycle conditions were 95°C for 30s, then 40 cycles of 95°C for 5 sec, 60°C for 34 sec. The qRT-PCR assays were conducted on an ABI 7500. Relative quantification (2^−ΔΔCt^) method was used for calculating fold changes. The Primer sequence for SNHG20, forward:5′-ATGGCTATAAATAGATACACGC-3′,reverse:5′-GGTAC AAACAGGGAGGGA-3′, for GAPDH, forward: 5′-CGG AGTCAACGGATTTGGTCGTAT-3′, reverse 5′-AGCCTT CTCCATGGTGGTGAAGAC-3′.

### RNA interference

Three siRNAs targeting SNHG20 and one negative control siRNA were purchased from Genepharma (Shanghai, China). In brief, 50 nM of each siRNA was transfected into 1 × 10 ^5^ MKN45 or BGC-823 cells per well in 6-well plate by using Lipofectamine 3000 Transfection Reagent (Invitrogen) according to the manufacturer's instructions. The sequences of siRNAs against SNHG20 were as follows: siSNHG20-1,sense, 5′-GCCUAGGAUCAUCCAGGUUTT-3′,anti-sense, 5′-A ACCUGGAUGAUCCUAGGCTT-3′; si-SNHG20-2, 5′-C CUGUUGUGUAUGGCUAUATT-3′, si-SNHG20-3, sense, 5′-UAUAGCCAUACACAACAGGTT-3′, anti-sense, 5′-GC CACUCACAAGAGUGUAUTT-3′. Si-EZH2, sense, 5′- TTCATGCAACACCCAACACT-3′, anti-sense, 5′- GAG AGCAGCAGCAAACTCCT-3′. Ectopic expression of SNHG20 was achieved through pcDNA3.1-SNHG20 transfection, with an empty pcDNA3.1 vector used as a control, the cells were harvested and detected after transfection at 48 h.

### Cell proliferation assay

The cell proliferation was measured by using Cell Counting Kit-8 (CCK-8) (Dojindo Molecular Technologies, Kumamoto, Japan). Briefly, cells transfected with si-SNHG20 or si-NC and pcDNA3.1-SNHG20 or pcDNA3.1 plasmid were seeded in a 96-well microplate with 3000 cells per well. Cells were cultured in 10% CCK-8 diluted in normal medium until visual color conversion occurred. Proliferation rates were detected at 0, 24, 48, 72 hours after transfection and measured in the absorbance at 450 nm.

### Cell migration and invasion assays

Cell migration and invasion were evaluated by using non-Matrigel-coated or Matrigel-coated transwell cell culture chambers (BD Matrigel Invasion Chamber; BD Biosciences, San Jose, CA, USA) of 8 μm pore size according to the manufacturer's instruction. For migration assays, 1 × 10^5^ cells were seeded in the top chamber without Matrigel (For invasion assay, the 1 × 10 ^5^ cells were seeded in the top chamber coated with Matrigel) supplemented with 200 μL serum-free medium, and 500 μL of 10% FBS containing medium was added to the lower chamber. After 24 hours, cells were fixed with methanol and stained with crystal violet. Cells on the upper surface were removed using a cotton swap. The number of migrating or invasion cells was calculated under the microscope in ten random fields and shown as the average per field.

### Western blotting analysis

Cells protein lysates were separated by 10% SDS-polyacrylamide gel electrophoresis (SDS-PAGE) and then transferred to nitrocellulose membrane. After blocking with 5% non-fat milk in TBST for 1 hour, membranes were incubated with primary antibody dissolved in 5% bovine serum albumin in TBST overnight at 4°C. The following primary antibodies were used: E-cadherin (1:1000, Cell Signaling Technology, USA), Vimentin (1:1000, Cell Signaling Technology, USA), Twist1 (1:1000; Cell Signaling Technology, USA) GSK-3β (1:3000; Abcam, USA), β-catenin (1:1000; Cell Signaling Technology, USA), p-GSK-3β (1:2000; Abcam, USA) and GAPDH (1:1000; Cell Signaling Technology, USA) was used as an internal control.

### RNA immunoprecipitation (RIP) assays

RIP experiments were performed according to previous report by using a Magna RIP RNA-Binding Protein Immunoprecipitation Kit (Millipore, USA) according to the manufacturer's instructions [13]. Antibody was used for RIP assays were EZH2, H3K27me3 (Cell Signaling Technology, USA). When RIP treated with RNase, lysates were incubated with RNase for 1 hour in 37°C. The co-precipitated RNAs were detected by RT-PCR analysis.

### Chromatin immunoprecipitation (ChIP) assay

ChIP assay was performed as previously described [14]. Briefly, Cell lysates were then sonicated to generate chromatin fragments and immunoprecipitated with EZH2 and H3K27me3-specific antibody (CST, USA) or IgG as control. Precipitated chromatin DNA was recovered and Real-time PCR was used to detect the amount of DNA immunoprecipitated by antibody.

### Statistical methods

The experiments were independently repeated at least triplicate times. Data were expressed as mean ± SD. Differences between two independent groups were tested with the student's test. All statistical analyses were performed by using SPSS 17.0 software. *P* < 0.05 was considered to be statistically significant.

## RESULTS

### Expression of SNHG20 is up-regulated in the GC cell lines and promotes the cell proliferation and invasion

**Figure 1:**
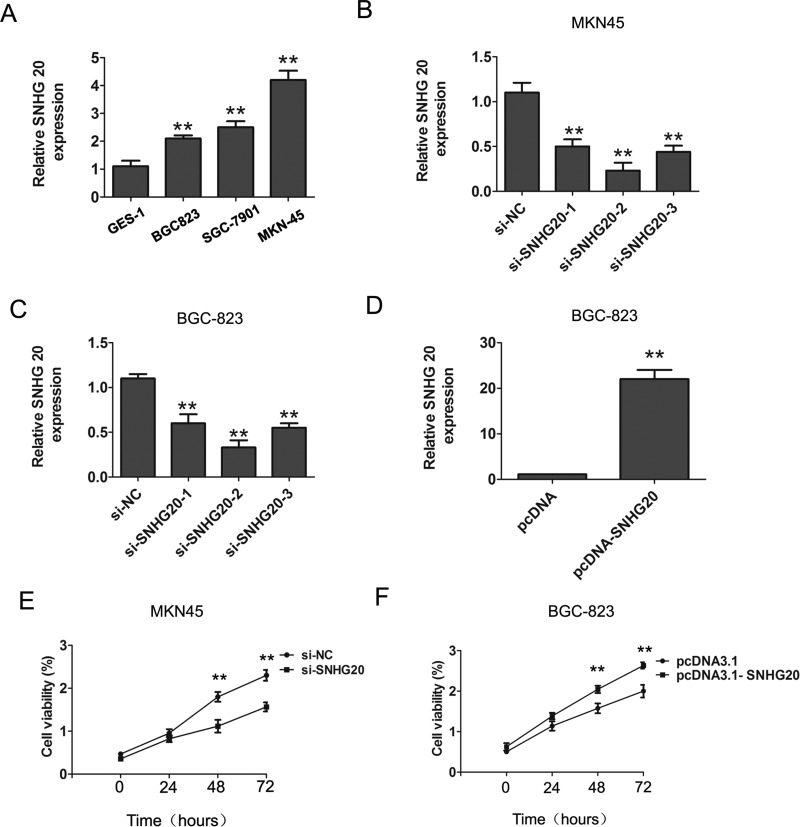
The increased expression of SNHG20 in GC cells (**A**)The relative expression of SNHG20 was detected in three GC cells (BGC-823, MKN45, SGC-7901) and an immortalized normal gastric epithelial cell line GES-1. SNHG20 expression was examined by qRT-PCR assays and normalized to GAPDH expression. (**B**–**C**) SNHG20 was knocked down by three si-SNHG20 oligos in MKN45 or BGC-823 cells. SNHG20 expression was examined by qRT-PCR analysis and normalized to GAPDH expression. (**D**) The SNHG20 was over-expression by transfecting pcDNA3.1 plasmid into BGC-823 cells. SNHG20 expression was examined by qRT-PCR analysis and normalized to GAPDH expression. (**E**) CCK8 assays were used to determine the cell proliferation ability after transfecting si-NC or si-SNHG20 into MKN45 cells. (**F**) CCK8 assays were used to determine the cell proliferation after transfecting pcDNA3.1 or pcDNA3.1-SNHG20 into BGC-823 cells, ** *P* < 0.05, Error bars represented the mean ± SD of triplicate experiments.

To explore the expression levels of SNHG20 in GC cells, we performed real-time PCR assay. The results showed that the expression of SNHG20 in three human GC cells were significantly up-regulated compared with normal gastric epithelial cell GES-1 (Figure 1A). To further detected the biological function of SNHG20 in GC progression, we knocked down SNHG20 in MKN45 or BGC-823 cells by using three siRNAs oligos (Figure 1B–1C), and overexpressed the SNHG20 levels by transfecting pcDNA3.1-SNHG20 into BGC-823 cells (Figure 1D).The results showed that the SNHG20 was effectively inhibited by si-SNHG20-2, and the si-SNHG20-2 was used in the following study.

**Figure 2:**
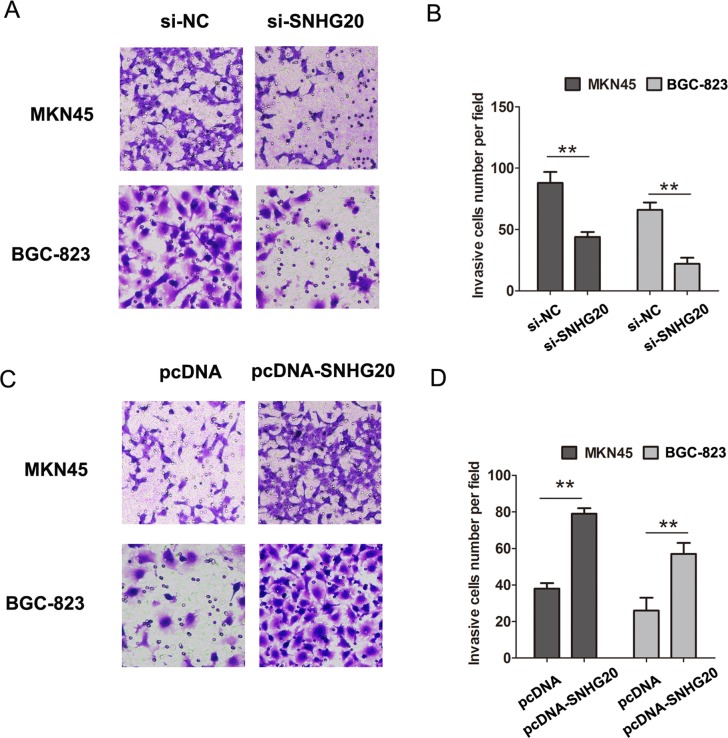
SNHG20 promoted the GC cell invasion in MKN45 or BGC-823 cells (**A**–**B**) Cell invasion abilities and analysis were evaluated by transwell invasion assays after transfecting si-NC or si-SNHG20 into MKN45 or BGC-823 cells. (**C**–**D**) Cell invasion abilities and analysis were evaluated by transwell invasion assays after transfecting pcDNA3.1 or pcDNA3.1-SNHG20 into MKN45 or BGC-823 cells, **P < 0.05, Error bars represented the mean ± SD of triplicate experiments.

To explore the role of SNHG20 on GC cell proliferation, the CCK8 cell proliferation assays were performed to evaluate the cell proliferation ability in MKN45 and BGC-823 cells. The results confirmed that knockdown of SNHG20 significantly inhibited cell proliferation in MKN45 cells, and overexpression of SNHG20 in BGC-823 cells promoted the cell proliferation (Figure 1E–1F). Moreover, after knockdown of SNHG20, the transwell cell invasion assays also demonstrated that the cells invasion abilities were markedly suppressed in MKN45 or BGC-823 cells (Figure 2A–2B), but was enhanced by ectopic expression of SNHG20 in MKN45 or BGC-823 cells (Figure 2C–2D). Together, these results demonstrated that SNHG20 could enhance gastric cancer cell proliferation and invasion *in vitro*.

### Over-expression of SNHG20 promotes the epithelial-mesenchymal transition (EMT) in GC cells

**Figure 3:**
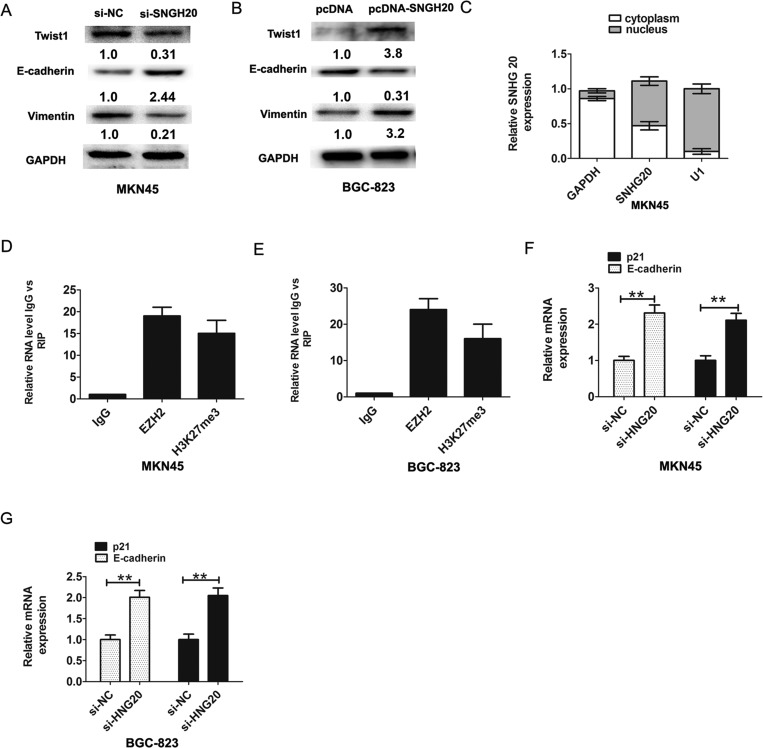
SNHG20 promoted GC cells epithelial-mesenchymal transition (EMT) (**A**) The relative protein expression of E-cadherin and Vimentin and Twist1 were detected by western-blotting analysis after knockdown of SNHG20 in MK45 cells and normalized to GAPDH expression. (**B**) The relative protein expression of E-cadherin and Vimentin and Twist1 were detected by western-blotting analysis after overexpression of SNHG20 in BGC-823 cells and normalized to GAPDH expression. (**C**) SNHG20 was enriched in cytoplasm and nucleus in MKN45 cells. The cytoplasm was normalized to GAPDH expression and nucleus was normalized to U1 expression. (**D**–**E**) RNA levels in immunoprecipitates with EZH2 and H3K27me3 antibodies were determined by qRT-PCR analysis in MKN45 or BGC-823 cells. Expression levels of SNHG20 were presented as fold enrichment relative to IgG immunoprecipitate. (**F**) The relative expression of E-cadherin and p21 was detected after knockdown of SNHG20 by qRT-PCR analysis in MKN45 cells, and normalized to GAPDH expression. (**G**) The relative expression of E-cadherin and p21 was detected after knockdown of SNHG20 by qRT-PCR analysis in BGC-823 cells, and normalized to GAPDH expression. **P < 0.05, Error bars represented the mean ± SD of triplicate experiments.

It is well recognized that EMT plays a critical role in GC invasion and metastasis [15]. Therefore, we further explored whether SNHG20 had effects on EMT phenomenon in GC cell. The western-blotting results showed that knockdown of SNHG20 significantly inhibited the expression levels of transcription factor Twist1 and EMT maker Vimentin, but upregulating the E-cadherin expression in MKN45 cells (Figure 3A). Ectopic expression of SNHG20 in BGC-823 cells promoted the expression levels of Twist1 and Vimentin, but downregulating the E-cadherin expression (Figure 3B). In general, these results showed that upregulation of SNHG20 promoted the epithelial-mesenchymal transition (EMT) in GC cells.

### SNHG20 interacts with EZH2 and inhibited the expression of E-cadherin and p21 in the GC cell

Recent evidences suggested that lncRNAs could regulate their target genes expression through interacting with RNA binding proteins [16]. Firstly, we analyzed the distribution of SNHG20 in GC cells. The results demonstrated that SNHG20 was existed in both cytoplasm and nucleus in MKN45 cells (Figure 3C). Furthermore, we performed RIP assays to detect SNHG20 whether directly binds to EZH2 and H3k27me3 in MKN45 or BGC823 cells, the RIP assays results confirmed that SNHG20 could bind to EZH2 and H3k27me3 in MKN45 or BGC-823 cells (Figure 3D–3E). These above data suggested that SNHG20 could epigenetically repress underlying targets expression at transcriptional level.

**Figure 4:**
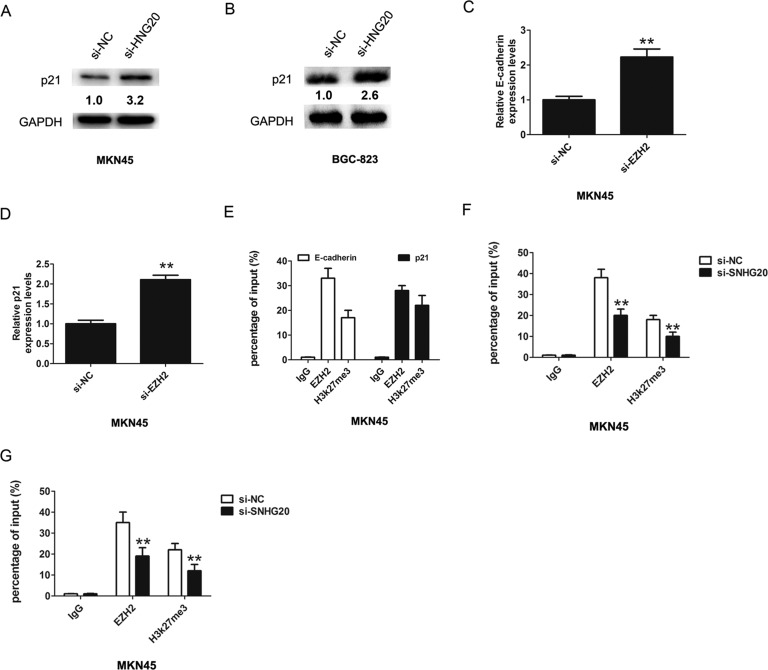
SNHG20 epigenetically silenced E-cadherin and p21 transcription by binding with EZH2 (**A**–**B**) The relative protein expression of p21 was detected after knockdown of SNHG20 by western-blotting in MKN45 or BGC-823 cells, and normalized to GAPDH expression. (**C**–**D**) The relative expression of E-cadherin and p21 was detected after knockdown of EZH2 by qRT-PCR in MKN45 cells, and normalized to GAPDH expression. (**E**) Chromatin immunoprecipitation-qPCR was used to analyze EZH2 occupancy, H3K27me3 binding to E-cadherin and p21 promoter regions in MKN45 cells. (**F**–**G**) Chromatin immunoprecipitation-qPCR was used to analyze EZH2 occupancy, H3K27me3 binding to E-cadherin and p21 promoter regions in MKN45 cells after knockdown of SNHG20. **P < 0.05, Error bars represented the mean ± SD of triplicate experiments.

EZH2 had been reported promoted tumor cell migration and invasion via epigenetic repression of E-cadherin [17]. In GC cells, a previous study found that long non-coding RNA ZFAS1 promoted gastric cancer cells proliferation by epigenetically repressing KLF2 and NKD2 expression [18]. Our results found that after knockdown of SNHG20, the p21 and E-cadherin expression were increased in MKN45 or BGC-823 cell (Figure 3F and Figure 3G). In addition, after knockdown of SNHG20, the protein levels of p21 were also increased in MKN45 and BGC-823 cells (Figure 4A–4B).

Furthermore, the mRNA levels of E-cadherin and p21 were also increased in MKN45 after knockdown of EZH2 (Figure 4C–4D). To confirm whether EZH2 could directly bind to the promoter region of p21 and E-cadherin, we constructed the primers across the promoter region. The ChIP assays revealed that EZH2 and H3k27me3 could bind to the p21 and E-cadherin (Figure 4E), and knockdown of SNHG20 reduced the EZH2 levels to the p21 and E-cadherin promoter region (Figure 4F–4G). Thus, these results demonstrated SNHG20 interacted with EZH2 and inhibited the expression of E-cadherin and p21 in the GC cell.

### SNHG20 regulated GSK-3β/β-catenin signaling pathway in the GC cell

**Figure 5:**
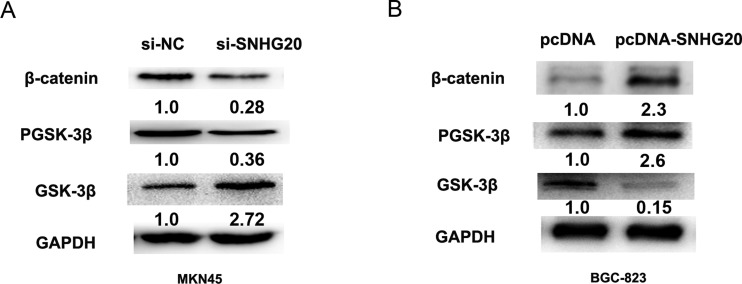
SNHG20 activated GSK-3β/β-catenin signaling pathway in the GC cell lines (**A**–**B**) The relative protein expression levels of GSK-3β, p-GSK-3β, β-catenin were detected after knockdown of SNHG20 or overexpression of SNHG20 by western-blotting analysis in MKN45 or BGC-823 cells, and normalized to GAPDH expression.

To investigate the effect of SNHG20 on Wnt/β-catenin signaling pathway, GSK-3β, p-GSK-3β and β-catenin were detected by the western-blotting analysis after knockdown or overexpression of SNHG20 in MKN45 or BGC-823 cells. As expected, the protein level of β-catenin and p-GSK-3β were significantly decreased, but GSK-3β was increased by transfecting si-SNHG20 into MKN45 cells (Figure 5A). However, the expression of β-catenin and p-GSK-3β was increased, but the GSK-3β was downregulated when transfecting pcDNA3.1-SNHG20 plasma into BGC-823 cells (Figure 5B). Thus, overexpression of SNHG20 activated GSK-3β/β-catenin signaling pathway in the GC cell.

## DISCUSSION

Recent findings have revealed that lncRNAs are involved in the development and progression of human disease, especially in some tumors [19]. However, the mechanism of action and biological function of SNHG20 in GC cells remain unknown. Only two reporters in previous study were involved in SNHG20. One is that Li *et al*. found that increased long non-coding RNA SNHG20 predicted poor prognosis of colorectal cancer [12]. The other study revealed that SNHG20 was overexpressed in HCC tissues and knockdown of SNHG20 in SK-Hep-1 cells significantly inhibited cellular proliferation, migration and invasion [11]. In the study, we demonstrated that the expression of SNHG20 was remarkably up-regulated in GC cells and knockdown of SNHG20 significantly inhibited GC cell proliferation, invasion and EMT.

LncRNAs could regulated cancer cells phenotypes through regulating target gene expression by different mechanisms, including chromatin modification, genomic imprinting, RNA decay and sponging miRNAs [20]. Such as, long non-coding RNA H19 increased bladder cancer metastasis by associating with EZH2 and inhibiting E-cadherin expression [21]. EZH2-mediated epigenetic suppression of long noncoding RNA SPRY4-IT1 promoted NSCLC cell proliferation and metastasis by affecting the epithelial-mesenchymal transition [17]. A recent study indicated that long non-coding RNA LINC01133 repressed KLF2, P21 and E-cadherin transcription through binding with EZH2, LSD1 in non small cell lung cancer [22]. Our results showed that knockdown of SNHG 20 inhibited the epithelial-mesenchymal transition (EMT), whereas, the overexpression of SNHG20 had the promoting effects. We further demonstrated that overexpression of SNHG20 significantly inhibited the expression of E-cadherin and p21 via binding to EZH2.

Aberrant activation of the Wnt/β-catenin signaling pathway has been demonstrated in several cancers, including GC cancer [23]. Numerous lncRNAs have been demonstrated to target the key components of the Wnt/β-catenin pathway and modulated cancer cell function. For example, H19 activated WNT signaling and promoted osteoblast differentiation by functioning as a competing endogenous RNA [24]. Long non-coding RNA CCAT2 promoted breast tumor growth by regulating the Wnt signaling pathway [25]. Our results demonstrated that overexpression of SNHG20 significantly regulated the GSK-3β/β-catenin signaling pathway.

In conclusion, our present results proved that SNHG20 plays vital roles in controlling GC cell invasion by activating EMT process. Furthermore, SNHG20 mediated the oncogenic effects through its epigenetic silencing of the p21 and E-cadherin expression by binding with EZH2 and regulated the GSK-3β/β-catenin signaling pathway. Thus, the results showed that inhibition of SNHG20 may serve as a therapeutic target for GC.
